# Micro”bee”ota: Honey Bee Normal Microbiota as a Part of Superorganism

**DOI:** 10.3390/microorganisms10122359

**Published:** 2022-11-29

**Authors:** Daniil Smutin, Egor Lebedev, Maxim Selitskiy, Nick Panyushev, Leonid Adonin

**Affiliations:** 1 Institute of Environmental and Agricultural Biology (X-BIO), Tyumen State University, 625003 Tyumen, Russia; 2 Department of Genetics and Biotechnology, Saint Petersburg State University, 199034 Saint Petersburg, Russia; 3 Group of Mechanisms for Nanosystems Targeted Delivery, Institute of Biomedical Chemistry, 119121 Moscow, Russia

**Keywords:** honey bee *Apis mellifera*, metagenome, hive, bacterial diversity, gut communities, symbiosis

## Abstract

Honey bees are model organisms for microbiota research. Gut microbiomes are very interesting for surveys due to their simple structure and relationship with hive production. Long-term studies reveal the gut microbiota patterns of various hive members, as well as the functions, sources, and interactions of the majority of its bacteria. But the fungal non-pathogenic part of gut microbiota is almost unexplored, likewise some other related microbiota. Honey bees, as superorganisms, interact with their own microorganisms, the microbial communities of food stores, hive surfaces, and other environments. Understanding microbiota diversity, its transition ways, and hive niche colonization control are necessary for understanding any separate microbiota niche because of their interplay. The long coevolution of bees with the microorganisms populating these niches makes these systems co-dependent, integrated, and stable. Interaction with the environment, hive, and other bees determines caste lifestyle as well as individual microbiota. In this article, we bring together studies on the microbiota of the western honey bee. We show a possible relationship between caste determination and microbiota composition. And what is primary: caste differentiation or microbiota composition?

## 1. Introduction

We now assume that all metazoans have symbiotic microbiota. It can affect any living process: metabolism, development, the work of defense systems, reproduction, and even speciation [1]. Balanced microbiota composition improves the health and productivity of the host. Vertebrates’ skin [2] and gut [3] microbiota are widely studied areas. High diversity and complexity, along with long coevolutionary processes, make these communities very interesting, but they are also very complicated to research. Often, revealing the role of single members in this community is almost impossible because their removal leads to total dysbiosis, and in vitro studies are affordable only for culturable microorganisms.

*Apis mellifera*, the honey bee, is a well-known model organism for studying microbiota. It represents a simple microbiome consisting of only a small subset of phylotypes and associated species, regardless of geographic location [4]. As in mammals, honey bee gut microbiota play a role in nutrition and digestion processes, protection against pathogens [5] and even bees’ behavior [6]. In contrast to mammalian microbiota, all members of the honey bee gut community were successfully cultured and can be used to colonize bees at germ-free stages [7].

**Figure 1:**
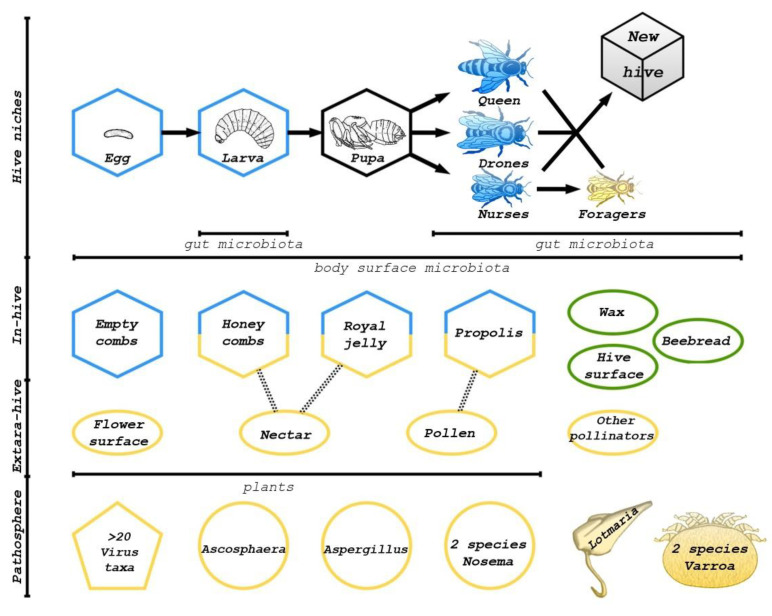
Types of honey bee-related microbiomes (according to [8,9,10]). There are 3 main groups of environments: bees, in-hive, and extra-hive. All microbiota there cannot strictly be divided to normal and pathogenic. Dotted lines depict the relationships between plant and in-hive microbiota. The developmental stages are linked by arrows. Ways of interaction and microorganism transmission are shown by color: blue for nurses, yellow for workers, and green for all hive members.

Honey bees are social insects with different castes: drones, queens, and workers (Figure 1). There are different interactions among them, and between them and preimaginal bees [8]. Interaction with the environment, hive, and other bees determines caste lifestyle as well as individual microbiota. While workers live in extra-hive environments their whole lives, queens leave the hive only to mate. Nurse bees’ roles are to feed the queen and brood. They also interact with other nestmates through oral trophallaxis (mouth-to-mouth feeding) and social grooming. Foodkeepers have fewer interactions with queens and larvae; their work is to store pollen and other resources from foragers. Foragers collect food from outside the hive and interact mainly with plants and other pollinators [9]. And what is primary: caste differentiation or microbiota composition?

## 2. Types of Honey Bee Microbiota

All types of honey bees’ related microbiota can be divided into several groups: gut symbiotic bacteria and fungi (which show differences between both life stages and castes); body surface microbiota (only partially studied); normal hive microorganisms in several ecological niches: honey combs, brood combs, their composing wax, propolis, and royal jelly; and the pathosphere (Figure 1).

In researching honey bees, the gut microbiota is the most studied part. In comparison with other animals, the honey bee’s gut microbiota has a very simple composition. Most bacteria live in the rectum and ileum, and 98% of their population belongs to 9 taxa (12 species) [11]. Most of the composition patterns and functions are performed by only five core species [12]. Non-core gut microbiota include several species from extra-hive environments with undetermined functions and values [10]. Diet, climate, and hive member caste all have a high impact on gut microbiota. By describing the honey bee hive as a superorganism, it shows changes in microbiota patterns in the area, but it is relatively stable over time, except for seasonal changes.

Any organism interacts with the environment and environmental bacteria through its surfaces. In contrast, these aspects of the microbiota have only been studied in part. Only a few articles analyze whole-body metagenomics data [10,13,14]. Therefore, the body surface microbiota of honey bees is almost completely unexplored.

Extra-hive environmental communities are deeply researched because of the high economic impact of related plants [15]. These studies are also significant because opportunistic environmental bacteria may be involved in gut microbiota disruption [12]. Moreover, these cenosis are the sources of some gut bacteria [16].

On the one hand, hive niches are intermediate between the environment and gut communities. Social interactions between hive members and stored food make these transitions possible. On the other hand, some microorganisms take part in honey, propolis, and royal jelly production, development of preimaginal stages. The pathosphere of honey bees includes more than 20 virus groups, mainly from Dicistroviridae and Iflaviridae [10,17,18]. There are also several pathogenic bacteria: *Melissococcus*, *Paenibacillus*, and *Spiroplasma* [19,20]. But the most harmful for colony health are the *Aspergillus*, *Nosema*, and *Varroa* species [10,13,20,21,22]. There are many good reviews about honey bee pathogens, so in this article we try to focus on the development and functions of normal microbiota and interactions among them, and between normal microbiota and pathogens.

## 3. Gut Microbiota

**Figure 2:**
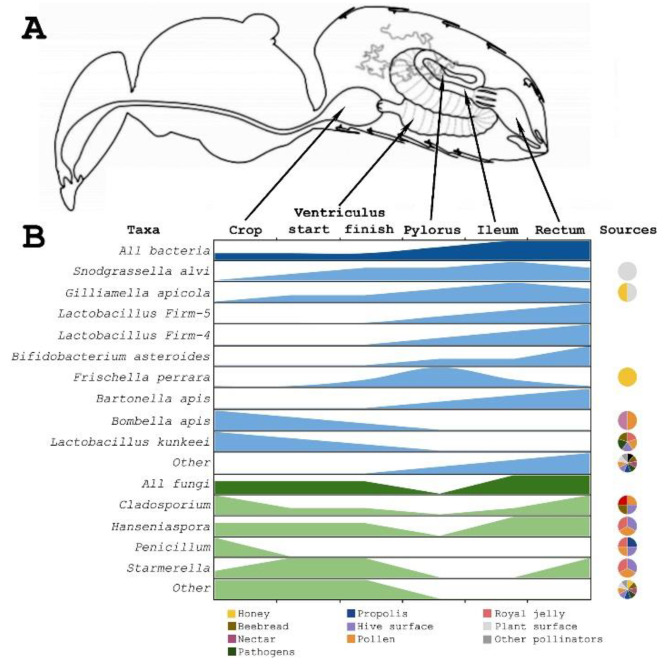
Amount and origins of main gut bacteria and fungi groups (according to [12,24,25,26,27,28,29,30,31,32,33,34,35,36,37]). Research on pylorus fungi has not been conducted yet. Number of microorganisms is provided on a relative scale. Probable environmental sources for community members are shown by color on pie charts on the right side.

Insects’ gut microbiota often represents simple communities with low diversity, large biomass, and a crucial role in nutrition processes. Only termites have different bacterial communities; in contrast, some bugs have only one core species [23]. Only five species’ clusters form the core of honey bees’ gut microbiome: *Snodgrassella alvi*, *Gilliamella apicola*, *Lactobacillus Firm-4*, *Lactobacillus Firm-5*, *Bifidobacterium asteroides*. Less numerous groups are: *Frischella perrara*, *Bartonella apis*, *Bombella apis* and *Commensalibacter* sp. (reviewed in [12]) (Figure 2).

There are differences between communities in different parts of the gastrointestinal tract. Workers’ stomachs and midguts are nearly devoid of bacteria. Their ileum and rectum are much more populated and contain nearly 95% of the total bacterial biomass [38]. The range of the primary gut bacterial groups’ roles may provide an explanation for their diversity (reviewed in [39]).

Crop communities are dominated by *Bombella apis* and *Lactobacillus kunkeei*, but total biomass of this organ is low [12]. *Bombella apis* was almost entirely found in honey bees and royal jelly, whereas *L. kunkeei* was found in flower cenosis [39]. These species are found by cultivating the crops of in-hive bees and honey, as well as by culture-dependent and culture-independent assessments of beebread [16,40]. As a result, some articles speculate on the crop community’s roles in beebread formation [41]. However, other research does not support that hypothesis [42].

The midgut also contains only a few bacteria. The community gradually changes along its length. *Snograssella alvi*, the core species, spreads evenly, while the *Giliamella apicola* biomass is closer to Pylorus. *S. alvi* and *G. apicola* are cross-feeding species. These species should be transmitted by contact with nurse bees or feces, but they are not found in honey bee hives, so they should not be transferred by oral trophallaxis like crop species [43] are. In addition, *Bombella apis* could be found there [40].

Pylorus is characterized by local colonization of *S. alvi* and non-core *F. perrara*. The *F. perrara* colonization provides scab formation [44] and causes some immune responses [45]. It transfers between hive members like the core *S. alvi* and *G. apicola* do. These bacteria often have different strains, even in one bee [46]. Special protein complexes (type VI secretion systems) have a high impact on their and some non-core species’ colonization of the gastrointestinal tract [47]. This system drives the evolution of toxin–antitoxin in bacteria–host interactions [48].

The ileum has a varied community in its invaginations and its lumen. There can be found co-living *S. alvi* in the lumen and *G. apicola* on the walls, where they form biofilm-like layers [40]. Members of the core groups *Lactobacillus Firm-4* and *Firm-5*, more widely presented in the rectum, can also be found in the ileum. Those strains have high diversity and have different metabolic interactions with their hosts [39]. *L. kunkeei* can be found only as part of the honey bee gut community because of their intolerance to atmospheric oxygen concentrations [12].

The rectum contains more firmicutes and actinobacteria than proteobacteria. *Lactobacillus firmicutes* types 5 and 4, as well as *B. asteroides*, are common in the rectum. A high degree of diversity in *Bifidobacterium* may be interpreted as various strains [49] or even several species [50]. The majority of them are only found in honey bee guts, but some strains can tolerate oxygen [51]. Specifically, many non-core bacteria can be found in the rectum, including *Bartonella apis*, *Commensalibacter* spp., and some other identified and several unidentified species [11,12,40,44].

*S. alvi*, *G. apicola*, *B. asteroides,* and *Lactobacillus Firm-4* and *Firm-5* occur in any worker [46]. Only as parts of gut communities were all core species found all over the world [38]. *S. alvi* and *Lactobacillus Firm-5* are found in bumble bees. These strains can also colonize honey bee guts, but with less efficiency in comparison to honey bee strains [52].

Other species exhibit diversity variations among hive members. According to the majority of researchers’ opinions, the main reason for the difference in native A. mellifera microbiota is the diet [53,54,55]. On the other hand, great influence on the non-core bacteria ratio and diversity is provided by social immunity factors and behavioral differences [43].

Core and some other bacteria are parts of the *A. mellifera* immune system [56,57]. *Lactobacillus* species, including the *Firm-5* phylotype, demonstrated in vitro and in vivo resistance to the pathogenic bacteria *Paenibacillus larvae* [58,59], as well as some other immune functions [60]. *G. apicola* and *S. alvi* biofilms are a protective layer against parasite invasion [46]. Also amazing is that engineered *S. alvi* can kill parasitic *Varroa* mites by triggering the mite RNAi response [20]. Non-core *Bombella apis* protects hives from fungal invasion [28,59]. *F. perrara* may also immunize bees [12].

In contrast to mammalian microbiota, the honey bee gut community was devoid of archaea and eukaryotes. Metagenomics revealed singularly matched archaeal 16S rRNAs and non-Apis 18S rRNAs [61,62]. Sometimes, during overwintering, some fungi occur in the ileum and rectum [63,64]. Their functions are still unknown, and their negative impact on health is accepted by a significant portion of researchers [36,63,64]. But other articles found a different fungal community along the digestive tract [34,37]. The amount of culturable fungi per organism is less than 10^4^ cells, in contrast to more than 10^8^ bacterial cells. Size correlations and possible interactions between fungal and non-core bacterial members were also discovered [34]. On the other hand, large differences between these communities may highlight fungi commensalism [37].

Gut microbiota changes during the development. Hatched and first instar larvae contain very low bacterial biomass. With aging, new bacteria occur in their guts: The 4th instar larvae community includes unknown *Rhizobiales* and *Fructobacillus* strains, *Lactobacillus* spp., *S. alvi*, *Bombella apis*, and rarely *F. perrara* [65,66,67]. Because these communities show significant differences between larvae from different hives, it is possible that this list does not include all taxa. Larval community patterns can be used as indicators of hive and queen health [61]. After that, the amount of bacteria in the larvae’s gastrointestinal tract drops to zero in pupae [12]. As a result, in the case of pupal dysbiosis, vertical transmission of gut microbiota between hives is also impossible.

Generative castes receive their gut microbiota from food, nurse bees, and in-hive environments. During the growing process, brood physiology, diet, and contacts with other bees gradually change [9,68]. All these factors and genetic differences drive microbiota stabilization processes. Young drones have gut microbiota very similar to workers [65]. In contrast, young queens’ gut is dominated by the *Escherichia* and *Enterobacteriales* species, and gradually, with development processes and the rise of interactions with workers and nurse bees, both their microbiota become more similar [69,70].

The process of workers’ gut colonization starts on the first day post-merger. Gut species have special orders and times of colonization [12,71]. Their ratio and diversity dynamically fluctuate. The amounts of *G. apicola*, *F. perrara*, *S. alvi,* and both core *Lactobacillus* groups are relatively stable, whereas the frequency of other species is variable. While colonization by the core species ends on the 3rd day post-merger, *L. kunkeei* populates the gut only until the 12th day [71].

In the long aging processes possible in nature, only the overwintering core microbiota show low variation, but other groups are unstable [14,64,72]. It is also related to the diet shift, so most of the non-core members not involved in metabolism disappear during overwintering [39,53,54,73]. Similar changes occur in hives in subtropical conditions [74]. Therefore, during the summer, non-core members can recover from non-hive environments or replenish their numbers through the acquisition of a single remaining cell. The details of these processes are still unclear.

It is interesting that when they coexist in biofilms, *G. apicola* and *S. alvi* have different fluctuations in the microbiota. While the amount of *G. apicola* consistently declines to its minimum level in October, the *S. alvi* biomass increases [42,65]. Similarly, *Lactobacillus Firm-5* and *F. perrara* levels continually decrease. It can be related to the immune response against these bacteria or the consequence of competition for their niches during the aging microbiota shift [54,74].

As it was mentioned above, most non-core members disappear from communities of winter bees. *Commensalibacter* spp. and *Bartonella apis* have aerobic respiration, while most other members are saccharolytic fermenters. It can be the reason for their biomass increasing during overwintering [39,54].

In different regions, trends in microbiota patterns and their dynamics are similar, but floral diversity and climate also matter [53,54,74,75]. Metagenomic surveys reveal correlations between honey bee gut microbiota and hive samples. Variation between hives cannot be explained only by geographic differences [75,76].

The process of microbiota colonization and variation is under the control of physicochemical properties, which can be modified by the host or the microbiome itself [34]. Conditions vary along the gut and during the bee’s life, which is reflected in space-specific and time-specific microbiota patterns [12,77]. That is also under immunity and social interaction control implemented in varied microbiota patterns among different hive members and hives [5].

## 4. Body Surface Microbiota

Body surface microbiota have a great impact on their hosts. It is well studied in humans [78] and other vertebrates [2]. Invertebrate surface microbiota are only partially studied. We assume two main reasons: on the one hand, gut microbiota are more stable than those on the body surface, and it is easy to avoid contamination during the sampling process. On the other hand, some groups such as insects show little surface biomass ([14,79,80], unpublished data), so cuticular microbiota identification becomes a very complicated and difficult process.

Cuticular microbiota of other ground Arthropoda have been described: spiders [81], caterpillars [82], ants [79,80], and recently—*Drosophila* [83]. Funnel-weaving spiders are model objects for studying surface bacteria–host interaction. Some results reveal even the behavior influence of cuticular bacteria [81]. Hosts should control surface microbiota, and cuticular microbiota could probably direct coevolutionary processes [83]. It is shown in ant populations that larger species ought to maintain more biomass and diversity in bacterial communities than smaller ones. It does not depend on DNA extraction methods and shows real situations (larger surface area sustains more niches) and less likely method limitations [79,80].

But the environmental microbiota associated with honey bees is predicted to contribute to the transformation, enhancement, and preservation of pollen and beebread, metabolic conversion, and nutritional status of bee products [84]. As it was mentioned above, few articles use whole-body metagenomics data [10,13]. Approximately 50–60% of OTUs are unrelated to gut microbiota. Therefore, the microbiota associated with other bee organs might also be different, but direct sequencing of bees without guts does not reveal any positive results [14,85].

Another way to research the body surface microbiota of *Apis mellifera* is through cultural methods [84,86]. Several articles report information about pure lines from honey bee broods. A recent study discovered 20 taxa of bacteria and yeasts on body surfaces [86]. According to the biomass, the main groups were: *Aureobasidium pullulans*, *Debaryomyces* spp., *Bacillus* spp., *Lactobacillus* spp., *Fructobacillus fructosus* and *Bifidobacterium asteroides*. These taxa are known as plant-related microflora. The role of major groups is still unknown. *Aureobasidium pullulans* is commonly used for plants’ pathogen biocontrol [87,88]. Therefore, we can suppose that some of these species might influence bees’ health and production.

## 5. In-Hive Environments Microbiota

Hive environments are, on the one hand, transition points for gut microbiota transfer [12,16,39,43,62]. On the other hand, hive products gradually change their biochemistry because of microbiota activity [16,89]. Microorganisms in the normal hive environment also protect hives from pathogens [90,91,92].

In hive environments, the core gut member *G. apicola* and several non-core members (Figure 2) can be found. *G. apicola* and *F. perrara* occur in bee bread and honey [24,25,93]. Their role in gut immunity is well-known, but their impact depends on bee age, metabolism, and own immunity [57,94]. Therefore, the influence of these bacteria on hive protection is unclear. The low level of their biomass does not allow us to assume metabolic functions [25]. Bee bread can be one of their transition ways, because they cannot be transmitted by oral trophallaxis [43].

*Bombella apis* and *L. kunkeei* are parts of the normal hive microflora and occupy almost all in-hive niches. In extra-hive environments, *Bombella apis* can be found in flower nectars [26,27,28,95], whereas *L. kunkeei* probably occupies more niches [16,31,33]. Therefore, the niche of *Bombella apis* indicates its role in extra-gut fermentation processes, larvae, and social immunity [28,95].

The microbiota of bee pollen differs between environmental pollen and gut microbiota [62]. Less than 10% of OTUs are similar between pollen and gut communities and might be residuals from the gastrointestinal tract [42,62]. The most abundant groups are *Firmicutes* [96], *Microbacteriaceae*, and *Enterobacteriaceae* [97]. Variations between samples might be the result of individual differences in the flight ways of workers [42]. Fresh pollen is non-consumable for honey bees, while fermented pollen is one of the main food sources for larvae [96,98]. On the other hand, distinct microbiota conserve pollen rather than convert it into new nutritional forms [29]. Because of *Bombella apis* presence or maybe other bacteria, bee pollen shapes the fitness of bee larvae [99,100].

Bee bread is produced by mixing nectar and bee pollen. It contains a more varied community than bee pollen [96], also because of more intensive fermentation processes. Key roles in this process are played by non-core *Lactobacillus* species [16,101]. Many extra-hive environmental organisms populate this niche, and *L. kunkeei* and *Bombella apis* also occur there. In bee bread and honey, other species are often detected by only sequencing or cultural methods, but not both of them [16].

Bee wax has antibacterial properties and, due to its composition, should not contain a different community [30,102], but as far as we know, no metagenomic research has been conducted on it.

Propolis is a product of partial bee wax fermentation by saliva and wax communities. It also has antimicrobial features [92], but another consistency makes it an important microbiota niche [33]. Its community is varied among hives [89]. Different groups dominate in different samples; the main ones are *Rhodopila* spp., *Corynebacterium* spp., *Sphingomonas* spp., *Erwinia* spp., and *Dickeya* spp. Most of these bacteria are saccharolytic microaerophiles. Propolis is also populated by different fungi. The fraction of dominant groups for both bacteria and fungi reaches 20–50% for one most abundant group and 50–95% for five of them. The most abundant groups are: *Candida* spp., *Aspergillus* spp., *Sydowia* spp., *Aureobasidium* spp., *Cladosporium* spp., and others [33,89]. In theory, the fungal community of worker crop has an origin mostly in propolis, except Hanseniaspora, but this requires more gut fungi research and correlation studies between fungal and propolis diversities. A species of plant should have a high impact on microbiota [103]. As we know by now, propolis fermentation is driven by non-gut bacteria, and saliva’s role in propolis formation is biochemical, but not microbiological. On the other hand, propolis ingestion stabilizes gut microbiota through its microorganisms and their biochemical compounds [104].

Honey appears to be one of the largest microbial communities [105]. Metagenomics shows that most of the honey diversity can be viral. In different samples, the most common was *A. mellifera* filamentous virus DNA [93]. Among bacteria, the most abundant group are *Bacillus* spp. [15] or *Lactobacillaceae* [93], mostly represented by fructophilic lactic acid bacteria [106]. As it was mentioned above, several other gut species also occur in honey. One article reports the presence of *B. asteroides* in honey [107]. They, *E. coli*, *Bacillus cereus*, *Salmonella enterica*, and some other microorganisms found in honey should play a role in its fermentation. *Zygosaccharomyces* sp. is a dominant fungus species [37,93,108]. Other sugar-concentration and ethanol tolerant yeasts, such as *Schizosaccharomyces* sp. and *Saccharomyces* sp., also occur here. On the one hand, honey is a source of plants’ and bees’ pathogens. On the other hand, several species, including *Penicillum* spp., *Pantoea agglomerans*, and *L. kunkeei*, might be a defensive layer against them [93]. The differences between samples are not as great as for beebread or propolis. Despite this, honey microbiota are regional and pollinated plant-specific, so they could be used to identify honey provenance [15,109,110,111]. Because empty combs and non-surface sterilized pupae contain the most similar microbiota [105], the hive should be one of the primary microorganism sources in honey.

One of the royal jelly metagenomes was used in the analysis of colony collapse disorder [112]. Another royal jelly metagenome is assembled but not analyzed yet [113], so this niche is only partially studied. The hindgut community, most similar to workers, was found in royal jelly, represented by non-core Xanthomonadaceae, *Bombella apis*, *L. kunkeei* and a few (~5% of total abundance) core gut bacteria, including *Lactobacillus Firm-5* and *Firm-4* bacteria, which are not found anywhere else except gut [42]. As a result, royal jelly may be a source of microbiota for its consumers. It is also known for its bactericidal properties, and it induces a social immunity response by transferring microbial pathogens between hives [90].

Hive surfaces contain all varieties of in-hive communities, but in small quantities. Despite this, its role in the transmission and control of pathogens should be important. Different *Aspergillus* spp. might be pathogenic or weaken the immune system, but their negative impact varies greatly [91]. The *Penicillum*, *Fusaria,* and *Ascosphaera* species were also found in hive environments [13]. Diverse fungi species have various influences; some of them seem to be “parasitic”, and some of them have no negative impact on honey bees’ health, so they can be commensal or even mutualistic to the hive as a superorganism [91].

## 6. Microbiota from the Environment, including Pathogens

Environmental microbiomes influence hive microbiota. Microorganisms are shared between plants and bees because of their contact in extra-hive environments and the transfer of plant resources to hives. The biochemistry of these sources controls their further colonization. Most of the non-core gut microbiota originate from nectar or pollen and normally occur in the air and on plant surfaces [15,114,115,116]. *L. kunkeei* has been found all over the world in different plant environments, especially pollen [31]. Metagenomics reveals the presence of core *Lactobacillus* species and non-core *Bifidobacterium* spp. and *Bombella apis* in nectars [29,116]. The big diversity of these groups may be related to different extra-hive environmental sources [49,66,117]. Therefore, bees may be considered as vectors for its transition, and plants as sources of normal gut microbiota [29]. Colonization of microbiota driven only by hive material leads to abnormal patterns in gut microbiota, when non-core taxa dominate in communities [43].

Plant surfaces, nectar, and pollen microbiota are widely researched [118,119,120]. Several articles try to track the origin of honey [109,110] and propolis [103], and they also analyze their natural sources. For these substrates, both biochemical properties and the microbial community itself control the process of later colonization and fermentation [104,105]. It is interesting that bacteria, not yeasts, can influence workers’ flower choices [121]. It might direct some co-specification processes among bacteria, bees, and flowers.

As it was mentioned above, several core species can be transferred between different pollinators. The role of this process in nature is unexplored. However, strains from different species have different specializations and have different colonization efficiencies [52].

Honey bee pathogens interact with normal microbiota and influence pattern differentiation [18,19,22]. Some species in the community could disappear, while others could increase their abundance and participate in the immune response [22,48,122]. During coinfection, there could be differences in the properties of their interactions [10].

Multicellular pathogens also share some microorganisms. *Varroa destructor* might be associated with bee pathogenic *Erwinia* sp., *Enterococcus faecalis*, *Stenotrophomonas maltophilia*, *Staphylococcus* spp., *Bacillus cereus,* and several other species [123,124]. Small hive beetles, *Aethina tumida*, are vectors for several normal gut species: *S. alvi, G. apicola*, *F. perrara*, and *L. kunkeei* [125]. They can also be transmitters of pathogens between hives [125,126].

But many interactions and the extent of the roles of non-gut microbial communities associated with honey bees are still unclear.

## 7. Conclusions

The honey bee gut microbiota is a model object to study host–bacteria interactions. Small diversity, high stability, and a set of composition patterns distinct due to caste, climate, and diet make various impact analyses and understanding the roles of different bacteria simple. Studies on various microbiota members allow one to figure out the metabolic role of bacteria under study, but often show similar results when researching their influence on honey bee fitness. It might indicate the complexity of their impact on development, reproduction, and defense system processes.

Almost always, all kinds of organism-related environments contain a great variety of microorganisms. Of course, the core whole-body microbiota has the greatest impact on the organism’s life. In contrast, other associated germs drive organism colonization processes and influence microbiota diversity and composition. Metabolic interactions are stronger between organisms’ gut microbiota, but food and body surface microbes also play a role in digestion and other biochemical processes. Therefore, both normal gut and environmental honey bee microbiota are parts of the *Apis mellifera* superorganism.

## Data Availability

Not applicable.
